# Oxidative Stress and Bone Resorption Interplay as a Possible Trigger for Postmenopausal Osteoporosis

**DOI:** 10.1155/2014/569563

**Published:** 2014-01-12

**Authors:** Carlo Cervellati, Gloria Bonaccorsi, Eleonora Cremonini, Arianna Romani, Enrica Fila, Maria Cristina Castaldini, Stefania Ferrazzini, Melchiorre Giganti, Leo Massari

**Affiliations:** ^1^Department of Biomedical and Specialist Surgical Sciences, Section of Medical Biochemistry, Molecular Biology and Genetics, University of Ferrara, Via Borsari 46, 44121 Ferrara, Italy; ^2^Department of Morphology, Surgery and Experimental Medicine, Menopause and Osteoporosis Centre, University of Ferrara, Via Boschetto 29, 44124 Ferrara, Italy; ^3^Department of Morphology, Surgery and Experimental Medicine, Laboratory of Nuclear Medicine, University of Ferrara, Via Aldo Moro 8, Cona, 44124 Ferrara, Italy; ^4^Department of Morphology, Surgery and Experimental Medicine, Section of Orthopaedic Clinic, University of Ferrara, Via Aldo Moro 8, Cona, 44124 Ferrara, Italy

## Abstract

The underlying mechanism in postmenopausal osteoporosis (PO) is an imbalance between bone resorption and formation. This study was conducted to investigate whether oxidative stress (OxS) might have a role in this derangement of bone homeostasis. In a sample of 167 postmenopausal women, we found that increased serum levels of a lipid peroxidation marker, hydroperoxides, were negatively and independently associated with decreased *bone mineral density* (BMD) in total body (*r* = −0.192, *P* < 0.05), lumbar spine (*r* = −0.282, *P* < 0.01), and total hip (*r* = −0.282, *P* < 0.05), as well as with increased bone resorption rate (*r* = 0.233, *P* < 0.05), as assessed by the serum concentration of C-terminal telopeptide of type I collagen (CTX-1). On the contrary, the OxS marker failed to be correlated with the serum levels of bone-specific alkaline phosphatase (BAP), that is, elective marker of bone formation. Importantly, multiple regression analysis revealed that hydroperoxides is a determinant factor for the statistical association between lumbar spine BMD and CTX-1 levels. Taken together, our data suggest that OxS might mediate, by enhancing bone resorption, the uncoupling of bone turnover that underlies PO development.

## 1. Introduction

Bone is a dynamic organ that undergoes continuous remodeling by the coordinated, and balanced, resorption and formation activities of, respectively, osteoclasts and osteoblasts [1]. The estrogen decline occurring in women after menopause frequently leads to a derangement of this homeostasis, with an increase of bone turnover rate and a state where resorption exceeds formation [2, 3]. These metabolic changes underlie the onset of postmenopausal osteoporosis (PO), a progressive disease characterized by low bone mass density (BMD), that predispose patients to an increased skeleton fragility and risk of fracture [2, 3]. Consistently, the *in vivo* determination of bone turnover is currently regarded as a helpful tool for the prediction of osteoporotic fractures and, mainly, for the monitoring of therapeutic efficacy [4]. Indeed, there are bone turnover markers that, reflecting the whole-body rates of either bone resorption or formation, provide reliable information regarding the health state of this tissue [4, 5].

In spite of the remarkable progresses achieved in the understanding of how estrogen deficiency induces PO, the underlying pathogenic mechanisms have been found to be complex and multifaceted [2]. One of the most intriguing hypothesis at this regard considers the ability of these sexual hormones to protect bone against oxidative stress (OxS) by acting as antioxidant [6]. *In vitro* and animal experiments, indeed, showed that estrogen withdrawal alters the generation of reactive oxygen species (ROS) and the antioxidant defense capacity of the cell [7], leading to an accumulation of these oxidant species, which, in turn, are able to stimulate osteoclast formation and resorption activity [8, 9].

This challenging body of evidence prompted us to investigate whether, also *in vivo*, OxS might be an influencing factor for the bone turnover impairment underlying PO development. To address this issue we evaluated a panel of distinct indicators of systemic OxS, along with marker of bone formation (bone-specific alkaline phosphatase, BAP) and resorption (C-terminal telopeptide of type I collagen, CTX-1) in a large population sample, including healthy, osteopenic and osteoporotic, postmenopausal women.

## 2. Materials and Methods

### 2.1. Subjects

The subjects examined in the present study were recruited among women undergoing bone densitometry evaluation at the Menopause and Osteoporosis Centre (MOC) of University of Ferrara (Ferrara, Italy). This study was carried out in accordance with the Declaration of Helsinki (World Medical Association, http://www.wma.net/) and the guidelines for Good Clinical Practice (European Medicines Agency, http://www.ema.europa.eu/) and it was approved by the Human research ethics committee of the University. Inclusion criteria were women in postmenopausal status, which was defined as cessation of menses for at least 1 year in accordance with the recent ReSTAGE's modification of the Stages of Reproductive Aging Workshop (STRAW) staging criteria [10]. Postmenopausal status was also checked by the assessment of follicle-stimulating hormone (FSH) and 17-*β* estradiol (E2) blood levels. According to a priori defined exclusion criteria, we excluded from the study those women who, while the study was being carried out, were using supplements containing the most common antioxidants such as vitamins E, C, and A, beta-carotene, and selenium or following vegetarian or vegan diet; drinking more than 20 g/day of alcohol; either affected by chronic diseases such as diabetes, malabsorption, and *cardiovascular disease* (CVD) or not diagnosed with a chronic disease, but taking medications (antiobesity medications, thyroid hormones, diuretics, antihypertensives, and anticholesterol drugs); undergoing hormone replacement therapy.

One hundred sixty-seven subjects were found to be eligible and were enrolled in the study after signing an informed consent. Each of these women underwent the measurement of body weight, standing height, and waist circumference by trained personnel. Fresh blood (7 mL) was drawn into Vacutainer tubes without anticoagulant by venipuncture after an overnight fast. After 30 minutes of incubation at room temperature, blood samples were centrifuged (4.650 g for 20 min), and the obtained sera were stored at −80°C until analysis.

### 2.2. Biochemical Assays

All the following assays were performed on serum samples using Tecan Sunrise-96 well microplate spectrophotometer (Tecan group Ltd., UK).

The levels of hydroperoxides were evaluated by colorimetric assay based on the reaction between these lipid peroxidation by-products and the chromogenic compound, that is, N,N-diethyl-para-phenylendiamine (from Sigma-Aldrich, St. Louis, MO, USA) [11–13]. Briefly, for each subject, 5 *μ*L of serum or standard (H_2_O_2_) was added to a solution containing 190 *μ*L of acetate buffer (pH 4.8) and 5 *μ*L of chromogen (0.0028 M). The solution was incubated at 37°C and then read for optical density after 1 and 4 minutes. The concentration of hydroperoxides was obtained by the average ΔA_505_/min and expressed as Carratelli Units (CU), where 1 CU corresponds to 0.023 mM of H_2_O_2_ [11, 12].

The concentration of advanced oxidation protein products (AOPP) was quantified as previously reported [14], with minor modifications. The AOPP assay includes a sample preparation procedure to precipitate triglicerides (3.000 g for 10 minutes in the presence of 25 mM/L MgCl_2_ and 0.5 mM/L phosphotungstic acid) which strongly interfere with the determination of the marker [14]. Subsequently, 30 *μ*L of supernatant serum (or the standard chloramine-T) was diluted 1 : 5 in phosphate-buffered saline. This solution was added into each well and mixed with 10 *μ*L of 1.16 M potassium iodide and 20 *μ*L of glacial acetic acid to each well. AOPP were measured at 340 nm and expressed as *μ*mol/L of chloramine-T (Sigma-Aldrich) equivalents [14].

The measurement of paraoxonase-1 (PON-1) basal activity was performed as described elsewhere [15]. After addition of 10 *μ*L of 3.3 mmol/L paraoxon (Sigma-Aldrich) to the assay mixture containing 5 *μ*L of serum and 2 mmol/L CaCl_2_ (in 100 mmol/L Tris/buffer, pH 8), to reach a final volume of 200 *μ*L, the formation of p-nitrophenol was monitored at 412 nm for 3 min. PON-1 basal activity was expressed as U/mL, where one unit is equivalent to 1 nmol of paraoxon hydrolysed/minute/mL.

Total concentration of thiols was determined by the colorimetric 5,5′-Dithiobis(2-nitrobenzoic acid)- (DTNB-) based assay described by Hu [16]. Serum (20 *μ*L), or standard (cysteine), was mixed with 160 *μ*L of 0.2 M Na_2_HPO_4_ and 2 mM EDTA at pH 8.0 into each well. The absorbance was determined at 405 nm, and 20 *μ*L of 10 mM DTNB (Sigma-Aldrich) in methanol was added to the sample. The absorbance obtained before the addition of DTNB was subtracted from that obtained after incubation with the chromogen. The concentration of thiol groups was expressed as *μ*moles/L.

The total concentration of nonenzymatic antioxidants (such as uric acid, ascorbic acid, and *α*-tocopherol) was determined by Ferric Reduction Antioxidant Power (FRAP) assay [17] with slight modifications [18]. FRAP method measures the ability of water- and fat-soluble antioxidants to reduce ferric-tripyridyltriazine (Fe^3+^-TPTZ) to the ferrous form (Fe^2+^) which absorbs at 593 nm. Briefly, acetate buffer (pH 3.6), TPTZ (10 mM), and FeCl_3_ (20 mM) were mixed in the ratio 10 : 1 : 1 to give the working solution. Serum (10 *μ*L), or standard (FeSO_4_), was added to 190 *μ*L of this solution. The reaction mixture was then incubated at room temperature for 6 minutes and the absorbance value was recorded at 595 nm. The results of this assay were expressed as FRAP units, where 1 FRAP unit corresponds to 100 *μ*moles/L of Fe^3+^ reduced to Fe^2+^ in 6 minutes.

Levels of ceruloplasmin (expressed as *μ*g/mL) were measured by quantitative competitive sandwich ELISA (AssayPro, St Charles, USA) according to the manufacturer's guidelines.

The measurement of BAP and CTX-1 concentration were performed using OCTEIA Ostase BAP immunoenzymometric assay and ß Cross-Laps Siero (CTx I), respectively, (both kits were from Immunodiagnostic Systems Ltd., Boldon, Tyne and Wear, UK) according to the manufacturer's guidelines.

Concentrations of E2 and FSH were determined by conventional chemiluminescent microparticle immunoassay using the commercial kits Architect Estradiol and Architect FSH from Abbot Laboratories (Abbott Park, IL, USA), respectively, according to the manufacturer's guidelines.

### 2.3. Bone Densitometry Assessment

Areal bone density was assessed at lumbar spine, hip, and total body by Discovery dual energy X-ray absorptiometry scanner (Hologic Inc, Bedford, MA). Postmenopausal osteoporosis was diagnosed when BMD *T*-score (the number of standard deviations below the average for a young adult at peak bone density) was lower than 2.5 standard deviations from BMD peak at either femoral neck or lumbar spine, according to WHO guidelines [19]. In accordance with these criteria, women with *T*-score at either skeleton area between −2.5 and −1.0 were classified as osteopenic and those with a value higher than −1.0 as normal.

### 2.4. Statistical Analysis

Data were analyzed using SPSS 18.0 for Windows (IBM, Chicago, IL, USA). Continuous variables were first analyzed for the normal distribution by the Kolmogorov-Smirnov and the Shapiro-Wilkinson test. Because the distribution of lumbar spine and neck BMD, CTX-1, hydroperoxides, AOPP, and thiols were highly skewed, we used their *base-10 logarithm* values as the outcome variables. One-way analysis of variance (ANOVA) and of covariance (ANCOVA) for unequal variances (implemented with Bonferroni *post hoc* test to compare two groups at a time) were used to evaluate the difference between sample groups before and after adjustment for confounding factors, respectively. Preliminary multiple regression analyses were performed to evaluate the possibility of collinearity problem among variables to include as covariates in multivariate analysis. Values of variance inflation factor (VIF) above 2.5 were regarded as indicative of multicollinearity. After this analysis, body mass index (BMI) was not included in the covariates set, because of its collinearity with waist circumference and of its weaker correlation with the variables of interest. Finally, univariate (by Pearson's correlation test) and multivariate (by partial correlation or multiple regression) analyses were performed to check the associations between continuous variables. A two-tailed probability value <0.05 was considered statistically significant.

## 3. Results

The characteristics of the 167 postmenopausal women enrolled in the present study are shown in Table 1. Women with PO were significantly (*P* < 0.01) older compared to those included in the other two study groups. Osteopenic and osteoporotic women presented lower mean values of years since menopause, BMI, and waist circumference compared to the healthy (*P* < 0.05 for all). On the contrary, frequency of smokers and serum levels of E2 and FSH did not significantly vary across the groups. By definition, total, neck, and lumbar spine BMD, as well as the correspondent *T*-score values, were significantly (*P* < 0.01) higher in healthy with respect to osteopenic and osteoporotic women. Finally, the levels of CTX-1 and BAP were not different among the groups.

As shown in Table 2, there were no significant differences in serum levels of OxS markers among the groups. However, osteopenia and osteoporosis appeared to be associated with a worse oxidative balance. Indeed, compared to healthy women, those affected by these two conditions presented higher levels of the lipid oxidative damage marker, hydroperoxides, and lower levels of total antioxidant power and ceruloplasmin.

**Figure 1:**
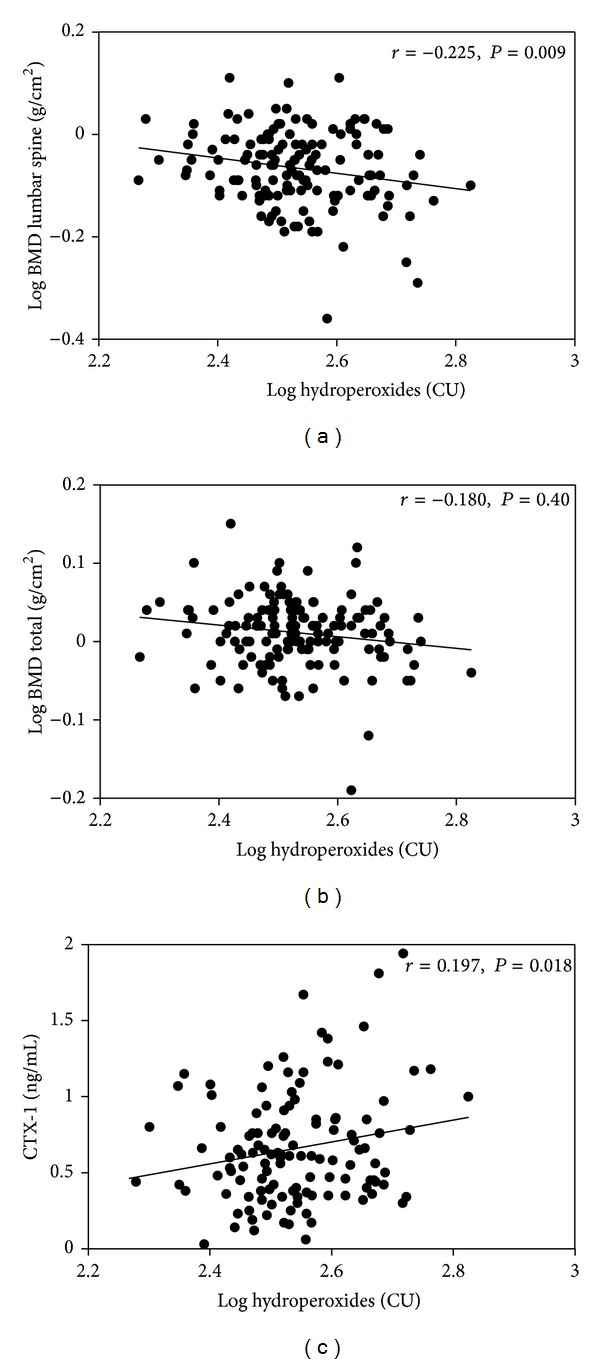
Scatterplots of the relationship between hydroperoxides and lumbar spine BMD (a), total body BMD (b), and CTX-1 (c) in the total sample (*n* = 167). Abbreviations: BMD: bone mass density; CTX-1: C-terminal telopeptide of type I collagen.

The possible association of OxS markers with levels of BMD and bone markers was initially checked by univariate analysis. Among the serum indicators of OxS considered in our study, only hydroperoxides showed significant associations with the parameters we used to evaluate bone health. In specific, this marker was found to be significantly associated with BMD at lumbar spine (*P* < 0.01), total body BMD (*P* < 0.05), and CTX-1 (*P* < 0.05) (Figures 1(a), 1(b), and 1(c), and Table 3). Moreover, these correlations remained significant after adjusting for potential confounding factors such as age, years since menopause, smoking, and waist circumference. Actually, as shown in Table 3, the strengths of the multivariate correlations between the OxS marker and the biochemical and densitometric bone parameters appeared to be stronger than the respective univariate. This effect was more evident for the correlation between hydroperoxides and total hip BMD that resulted to be significant (*P* < 0.05) only in the multivariate analysis.

Since higher levels of CTX-1 and hydroperoxides were found to be predictors of lower lumbar spine BMD, multiple regression analyses were run to unveil whether these associations were independent to each other. To this aim, three separate multiple regression models were performed, where each one included age, years since menopause, smoking, and waist circumference plus the following: hydroperoxides (model 1), CTX-1 (model 2), and hydroperoxides and CTX-1 (model 3). As displayed in Table 4,* the correlation between hydroperoxides and lumbar spine BMD* was significant (*P* < 0.01) regardless of the presence of CTX-1 among the covariates. On the contrary, the association between CTX-1 and lumbar spine BMD did not persist when the OxS marker was included in the multivariate model.

## 4. Discussion

The widely accepted concept of OxS as a condition that is mutually correlated with aging [20, 21] *has* represented the rationale of several studies on the link between accumulation of oxidative damage to biomolecules and the onset of PO [6, 22, 23]. Actually, although PO is a common disease of the elderly, *its main initiating factor is the menopause-related estrogen decline rather than the aging* [3]. It is widely accepted, indeed, that these sexual hormones can protect women against bone loss during the reproductive age [2, 3]. Several lines of evidence suggest that one of the mechanisms adopted by estrogens to accomplish this aim consists of contrasting the, supposed deleterious action of ROS against bone health [2, 6–8]. The data in support of the interplay between OxS and PO onset have been mostly obtained from *in vitro* and animal experiments, which, overall, suggest a potential role of reactive species in uncoupling bone turnover [7, 8, 23]. However, the definitive consensus on the involvement of OxS in the derangement of bone homeostasis is still lacking, due to the controversial results of the few *in vivo* human studies so far conducted.

The present cross-sectional population-based study shows that PO and osteopenia are not associated with an evident impairment of systemic oxidative balance. Indeed, the observed trend of oxidative damage (hydroperoxides and AOPP) and antioxidant defence (thiols, total antioxidant power, PON-1, and ceruloplasmin) markers to increase and decrease, respectively, in subjects affected by these two conditions was not statistically significant. On the other hand, high levels of one of the markers examined, hydroperoxides, were significantly, and independently of potential confounding factors, associated with low BMD in two districts of skeleton, total hip, and lumbar spine, that are highly susceptible to PO-related fractures. These outcomes were in line with the previous few studies involving a homogenous postmenopausal female population [23–25] as ours, but also more heterogeneous populations of men and women [26, 27] or only of men [28]. The aforementioned negative correlation between hydroperoxides and BMD was not reflected in a significant increase of this marker in PO patients most probably because the diagnosis of this bone disease is conventionally based upon *T*-score, a parameter that is statistically derived from BMD, that, indeed, was not found to be associated with hydroperoxides. Moreover, detectable alterations in circulatory levels of OxS markers are mainly associated with diseases, where, differently from PO, there is an intense tissue damage with consequent release of prooxidant metal ions like iron and copper and mitochondrial impairment or in chronic metabolic disorders such as diabetes and vascular chronic inflammation [21, 29]. Thus, in line with previous studies [6, 25, 30], our results suggest that systemic OxS, *even if* it could not be by itself a distinctive condition of women with PO, *negatively affects bone health thereby increasing the risk to develop this bone degenerative disease*.

Among the findings of our study, the significant positive association between serum levels of hydroperoxides and CTX-1, that is, marker of bone resorption, was the one that mostly adds to the current literature. Indeed, to the best of our knowledge, only another population-based study found this association among postmenopausal women, by using, however, different markers for OxS, that is, 8-hydroxy-2′-deoxyguanosine, and bone resorption, that is, cross-linked carboxyterminal telopeptide of type I collagen (ICTP). These methodological differences between the two works are not entirely negligible, mostly in relation to the latter marker. Indeed, as nicely described by Garnero et al. [31] and others [32, 33], ICTP and CTX-1 reflect different collagenolytic pathways, which in turn take place in distinct bone pathologies. When compared to the other markers, CTX-1 serum level was showed to *be* a more reliable indicator of the osteoclastic resorptive activity. Consistently, the assessment of CTX-1, but not of ICTP, is recommended for monitoring the effectiveness of antiresorptive therapy in patients affected by PO [31].

Further insight into the understanding of the “weight” of OxS in bone loss of postmenopausal women was provided by multivariate analysis. As shown in Table 4, the relationship between decrease in lumbar spine BMD and increase in CTX-1 serum levels was obvious after taking into account a set of strong potential predictors of bone loss (i.e., smoking, age, years since menopause, and waist circumference), but it disappeared after including also hydroperoxides in this set. This statistical outcome led us to speculate that the degradation of the main collagen component of bone organic matrix might be, at some extent, dependent on OxS. This hypothesis is vastly supported by previous *in vitro* studies showing that ROS stimulates osteoclast differentiation and bone resorption in mouse calvarial and bone marrow cultures [34, 35] and cocultures of mouse calvarial osteoblasts and spleen cells [36]. More recently, it has been shown that the increase of ROS, *mostly due to xanthine/xanthine oxidase activity* [37], stimulates the resorption process by triggering osteoclastogenic Nuclear Factor-kappa *β* (NF-*κβ*) ligand (RANKL)-RANK signaling between osteoblasts and osteoclast precursors [9, 37]. RANKL binding to RANK initiates osteoclast differentiation and activation and is critical for maintaining their survival and for promoting bone resorption [37]. Consistently, experiments on human bone marrow cells demonstrated that *hydrogen* peroxide is able to induce the expression of RANKL as well as macrophage colony stimulating factor (M-CSF), which activates the former by inducing the expression of RANK on myeloid cells.

Finally, it is fair to acknowledge that the cross-sectional nature of the present study limits our ability to establish any cause-effect relationship between OxS, CTX-1, and BMD. *However, our observations, although preliminary, may represent an important basis for a future longitudinal study aimed to evaluate the potential beneficial effects of nutritional antioxidants on bone health*.

## 5. Conclusion

In conclusion, our findings show an association between increased hydroperoxides serum levels and reduced bone density in postmenopausal women. Besides, this is the first population-based study showing a positive independent association between this lipid peroxidation marker and serum levels of CTX-1. These results would suggest that OxS might play a role in the development of PO *by* enhancing bone resorption rate. Additional studies are warranted to definitely establish a causal relationship between OxS and bone loss in postmenopause.

## Figures and Tables

**Table 1:** Principal characteristics of healthy, osteopenic, and osteoporotic postmenopausal women.

	Healthy (*n* = 38)	Osteopenia (*n* = 73)	Osteoporosis (*n* = 56)
Age, years	53.7 ± 4.6	55.6 ± 4.5	58.4 ± 4.3^a,b^
Years since menopause, years	7.4 ± 0.8	7.0 ± 0.8^a^	6.9 ± 0.7^a^
BMI, kg/m^2^	26.4 ± 4.1	24.4 ± 2.9^a^	24.2 ± 3.2^a^
Waist circumference, cm	89.3 ± 9.6	84.0 ± 9.2^a^	83.1 ± 8.4^a^
Smoking, %	17.8	14.7	12.9
E2, pg/mL	21.1 ± 7.1	12.9 ± 1.8	13.8 ± 2.6
FSH, mIU/mL	72.1 ± 7.2	85.2± 4.2	79.2 ± 5.2
DXA parameters			
Lumbar spine BMD, g/cm^2^	1.04 ± 0.09	0.88 ± 0.09^a^	0.75 ± 0.08^a,b^
Lumbar spine *T*-score	−0.09 ± 0.77	−1.46 ± 0.73^a^	−2.77 ± 0.70^a,b^
Femoral neck BMD, g/cm^2^	0.81 ± 0.06	0.69 ± 0.06^a^	0.62 ± 0.08^a,b^
Femoral neck *T*-score	−0.28 ± 0.59	−1.39 ± 0.6 ^a^	−2.06 ± 0.74^a,b^
Total hip BMD, g/cm^2^	0.90 ± 0.06	0.81 ± 0.07^a^	0.62 ± 0.07^a,b^
Total hip *T*-score	−0.21 ± 0.51	−1.04 ± 0.66^a^	−1.72 ± 0.62^a,b^
Total body BMD, g/cm^2^	1.09 ± 0.16	1.05 ± 0.07^a^	0.95 ± 0.07^a,b^
Total *T*-score	0.35 ± 0.99	−0.67 ± 0.68^a^	−1.60 ± 0.74^a,b^
Bone markers			
CTX-1, ng/mL	0.60 ± 0.21	0.66 ± 0.39	0.67 ± 0.40
BAP, *µ*g/L	27.7 ± 2.7	25.7 ± 1.2	25.1 ± 1.7

Data presented are expressed as % within the group for categorical and mean ± standard deviations for continuous variables.

^a^ *P* < 0.05 versus healthy; ^b^ *P* < 0.05 versus osteopenia.

Abbreviations: BMI: body mass index; E2: estradiol; FSH: follicle stimulating hormones; BMD: bone mass density; CTX-1: C-terminal telopeptide of type I collagen; BAP: bone-specific alkaline phosphatase.

**Table 2:** OxS markers mean levels in healthy, osteopenic, and osteoporotic postmenopausal women.

	Healthy (*n* = 38)	Osteopenia (*n* = 73)	Osteoporosis (*n* = 56)
Hydroperoxides (CU)	349.3 ± 12.3	352.7 ± 11.7	370.6 ± 10.8
AOPP (*μ*moles/L)	82.1 ± 8.8	76.6 ± 2.9	87.4 ± 7.6
Thiols (*μ*moles/L)	225.9 ± 13.9	215.2 ± 12.7	225.0 ± 17.3
Total antioxidant power (FRAP units)	734.1 ± 28.2	675.7 ± 15.7	697.2 ± 21.6
PON-1 (U/mL)	134.1 ± 9.2	138.2 ± 9.3	122.1 ± 12.2
Ceruloplasmin (mg/dL)	52.6 ± 6.7	49.2 ± 4.0	45.9 ± 5.1

Data presented are expressed as mean ± standard errors for continuous variables.

Abbreviations: AOPP: advanced oxidation protein products; CU: Carratelli Units; FRAP: Ferric reduction antioxidant capacity; PON-1: paroxonase-1.

**Table 3:** Simple and partial correlation coefficients for the association of hydroperoxides with total, lumbar spine, and total hip BMD, as well as CTX-1 in the total sample (*n* = 167).

	Simple correlation (*r*)	Partial correlation (*r*)
Lumbar spine BMD	−0.225^b^	−0.282^b^
Total hip BMD	−0.120	−0.208^a^
Total body BMD	−0.180^a^	−0.192^a^
CTX-1	0.197^a^	0.233^a^

^a^ *P* < 0.05, ^b^ *P* < 0.01.

Adjusting variables for partial correlation: age, years since menopause, smoking, and waist circumference.

Abbreviations: BMD: bone mass density; CTX-1: C-terminal telopeptide of type I collage.

**Table 4:** Multiple regression analysis of the association of hydroperoxides and CTX-1 with lumbar spine BMD.

Explanatory variables		
	B* (SE)	*β* ^ #^
Model 1		
Hydroperoxides	0.262^b ^ (0.061)	−0.321

	Adjusted *R* ^2^ = 0.25	

Model 2		
CTX-1	−0.044^a ^ (0.023)	−0.172

	Adjusted *R* ^2^ = 0.16	

Model 3		
Hydroperoxides	−0.246^b ^ (0.066)	−0.301
CTX-1	−0.026^ ^ (0.023)	−0.100

	Adjusted *R* ^2^ = 0.25	

Model 1: age, years since menopause, smoking, waist circumference + hydroperoxides.

Model 2: age, years since menopause, smoking, waist circumference + CTX-1.

Model 3: age, years since menopause, smoking, waist circumference + hydroperoxides and CTX-1.

^a^ *P* < 0.05; ^b^ *P* < 0.01.

*Unstandardized regression coefficient; ^#^standardized regression coefficient.

Abbreviations: SE: standard error; CTX-1: C-terminal telopeptide of type I collagen.
